# Cost-effectiveness analysis of diarrhoea management approaches in Nigeria: A decision analytical model

**DOI:** 10.1371/journal.pntd.0006124

**Published:** 2017-12-19

**Authors:** Charles E. Okafor, Obinna I. Ekwunife

**Affiliations:** Department of Clinical Pharmacy and Pharmacy Management, Faculty of Pharmaceutical Sciences, Nnamdi Azikiwe University Awka, PMB 5025, Nigeria; Oxford University Clinical Research Unit, VIET NAM

## Abstract

**Background:**

Diarrhoea is a leading cause of death in Nigerian children under 5 years. Implementing the most cost-effective approach to diarrhoea management in Nigeria will help optimize health care resources allocation. This study evaluated the cost-effectiveness of various approaches to diarrhoea management namely: the ‘no treatment’ approach (NT); the preventive approach with rotavirus vaccine; the integrated management of childhood illness for diarrhoea approach (IMCI); and rotavirus vaccine plus integrated management of childhood illness for diarrhoea approach (rotavirus vaccine + IMCI).

**Methods:**

Markov cohort model conducted from the payer’s perspective was used to calculate the cost-effectiveness of the four interventions. The markov model simulated a life cycle of 260 weeks for 33 million children under five years at risk of having diarrhoea (well state). Disability adjusted life years (DALYs) averted was used to quantify clinical outcome. Incremental cost-effectiveness ratio (ICER) served as measure of cost-effectiveness.

**Results:**

Based on cost-effectiveness threshold of $2,177.99 (i.e. representing Nigerian GDP/capita), all the approaches were very cost-effective but rotavirus vaccine approach was dominated. While IMCI has the lowest ICER of $4.6/DALY averted, the addition of rotavirus vaccine was cost-effective with an ICER of $80.1/DALY averted. Rotavirus vaccine alone was less efficient in optimizing health care resource allocation.

**Conclusion:**

Rotavirus vaccine + IMCI approach was the most cost-effective approach to childhood diarrhoea management. Its awareness and practice should be promoted in Nigeria. Addition of rotavirus vaccine should be considered for inclusion in the national programme of immunization. Although our findings suggest that addition of rotavirus vaccine to IMCI for diarrhoea is cost-effective, there may be need for further vaccine demonstration studies or real life studies to establish the cost-effectiveness of the vaccine in Nigeria.

## Author summary

‘‘Cost-effectiveness analysis of diarrhoea management approaches in Nigeria: a decision analytical model” was an original research carried out due to the high prevalence and mortality rate due to diarrhoea in Nigeria. The study aims to determine which treatment approach for diarrhoea would be cost-effective for Nigerians to implement due to limited resources available. Rotavirus vaccine plus integrated management of childhood illness for diarrhoea approach (rotavirus vaccine + IMCI) was the most cost-effective treatment approach.

## Introduction

### Background

Globally, diarrhoea is the second leading cause of death in children under 5 years [1]. There are about 1.7 billion annual cases of diarrhoea in the world and 760,000 annual deaths of children under 5 years due to diarrhoea [2]. In Nigeria, 11% of childhood deaths are caused by diarrhoea while only 1% of these children with diarrhoea receive the right treatment [3]. Diarrhoea kills over 90,900 children under the age of 5 yearly in Nigeria, which translates to about 249 deaths daily [3].

A plausible cause of the high diarrhoeal mortality could be the use of wrong medications. These medications include anti-motility agents (e.g. loperamide or tincture of opium), adsorbents (e.g. kaolin & pectin) and antibiotic (e.g. co-trimoxazole, ciprofloxacin). In Nigeria for instance, more than 62% of caregivers were prescribed antibiotics while 35% prescribed anti-motility drugs for childhood for diarrhoea [4]. Anti-motility agents are not suitable for childhood diarrhoea because they are ineffective in treating the pathogenic causes of diarrhoea and can cause partial or complete blockage of the bowel, resulting in an inability to pass stool and lethargy [5]. They do not prevent dehydration and their mode of action can lead to build-up of pathogenic toxins in the intestine and make the illness last longer [6]. The World Health Organization (WHO) and United Nations International Children’s Emergency Fund strongly discourage its use in infants and children [6]. Similarly, adsorbents attract water, toxins and bacteria from the digestive tract [7]. They do not help to replenish the water and electrolyte that are lost and they have not been proven safe and efficacious in cases of paediatric diarrhoea.

The right approach to management of childhood diarrhoea includes prevention through rotavirus vaccine and use of oral rehydration salt (ORS) or Intravenous fluid (IVF). Rotavirus vaccine protects against rotavirus infections, the leading cause of severe diarrhoea among young children [8]. The vaccine prevents 15 to 34% of severe diarrhoea in the developing world and 37 to 96% of severe diarrhoea in the developed world [9]. The vaccine is recommended by WHO for inclusion in national programme of immunization especially for diarrhoea endemic countries [9]. Low osmolarity ORS plus zinc are recommended by the WHO as the first-line treatment for childhood diarrhoea [10], and is the adopted approach for diarrhoea management under the integrated management of childhood illness (IMCI). ORS plus zinc have been proven to speed recovery, restore strength, energy and appetite and help keep children thriving [11], [12]. WHO also recommend the inclusion of breastfeeding and retinol to the management approach [13], [14]. Although the inclusion of zinc to ORS in the management is gaining acceptance in Nigeria, the inclusion of retinol and breast feeding practices are still poor [15].

Most recent studies on cost-effectiveness analysis of diarrhoea management using the right treatment approaches failed to reflect the combined effect of other components of IMCI for diarrhoea, i.e. breastfeeding, zinc tablets and retinol in addition to ORS or IVF. They considered zinc alone [16], ORS alone, ORS plus zinc alone [17], [18], or ORS plus rotavirus vaccine alone [19]. It is necessary to evaluate the holistic effect of these components in a cost-effectiveness analysis. Furthermore, to the best of our knowledge, no study on cost-effectiveness of diarrhoea management in Nigeria has been conducted. As a disease with high prevalence in Nigeria, there is need to evaluate the best approach to its management.

### Objectives

This study evaluated the cost-effectiveness of various approaches to diarrhoea management namely: the ‘no treatment’ (NT) approach; the preventive approach with rotavirus vaccine; the IMCI approach; and rotavirus vaccine + IMCI approach.

## Methods

### Study setting and target population

Participants were an estimated population of 33 million Nigerian children under five years at risk of having diarrhoea (well state). This figure was based on the 2016 population report which indicated that about 18% of Nigerians are between 0–5 years and this group are at risk of having diarrhoea [20].

The study was carried out in Nigeria using a decision analytic model (Markov cohort model) and depicted the Nigerian scenario. The model incorporated data to simulate a real life scenario.

### Study perspective

This simulation based decision analytic Markov model used retrospective data to compare four treatment approaches. Cost was estimated from the payer’s perspective (e.g. Health Maintenance Organisations, Government, Insurance companies etc). Cost items involved in this perspective included direct medical cost [21].

### Interventions description

Diarrhoea management approaches or interventions considered in this decision analytic model were based on the recommendation of the WHO [9], [10]. The specific details of these approaches are shown in Table 1. The four competing approaches in the decision analysis include:

1. The NT approach–In this approach, only routine breastfeeding was given to the child. This approach was the base case comparator.
2. The rotavirus vaccine approach–Rotavirus vaccine was administered between the ages of 6 weeks to 32 weeks in two doses. The first dose was administered between the ages of 6 to 15 weeks while the second dose was administered not later than 32 weeks [9]. Routine breastfeeding was also included in this scenario. Our analysis was based on the live monovalent human attenuated rotavirus vaccine (RV1).
3. The IMCI approach—In addition to routine breastfeeding, zinc, retinol and low osmolarity ORS was administered for moderate diarrhoea while zinc, ringers lactate IVF and retinol was administered for severe diarrhoea.
4. The rotavirus vaccine + IMCI approach—This involves vaccination with RV1 and management of diarrhoea as recommended by IMCI.

**Table 1:** Diarrhoea management approaches.

Approach	Interventions	Dosage	Duration	Source
**No treatment**				
	Breastfeeding	Q.S.	52 weeks	[13]
**Rota virus vaccine** **(70% coverage)**				
	Breastfeeding	Q.S.	52 weeks	[13]
	Rotavirus vaccine	1ml	32 weeks (2 doses)	[9]
**IMCI diarrhoea**				
Moderate Diarrhoea	Breastfeeding	Q.S.	52 weeks	[13]
	L-ORS		Typically 3 days/episode	[6], [10]
	Zinc supplement	10mg/day or 20mg/day	10 days/episode	[6], [10]
Severe Diarrhoea	Breastfeeding	Q.S.	52 weeks	(13)
	IVF (Ringer’s Lactate)	10 – 20ml/kg/hr.	Typically 3 days/episode	[6], [10]
	Zinc supplement	10mg, 20mg	10 days/episode	[6], [10]
	Retinol	25 – 100ml (50,000IU– 200,000IU)	6 months	[13], [14]
**Rota virus vaccine + IMCI diarrhoea**				
	Rotavirus vaccine (RV1)	1ml		[9]
Moderate diarrhoea	Rotavirus vaccine (RV1) plus IMCI as above	See above		See above
Severe diarrhoea	Rotavirus vaccine (RV1) plus IMCI as above	See above		See above

Q.S: Sufficient Quantity; L-ORS: Low osmolarity Oral Rehydration Salt

IMCI: Integrated Management of Childhood Illness

### Choice of model and assumptions

**Fig 1:**
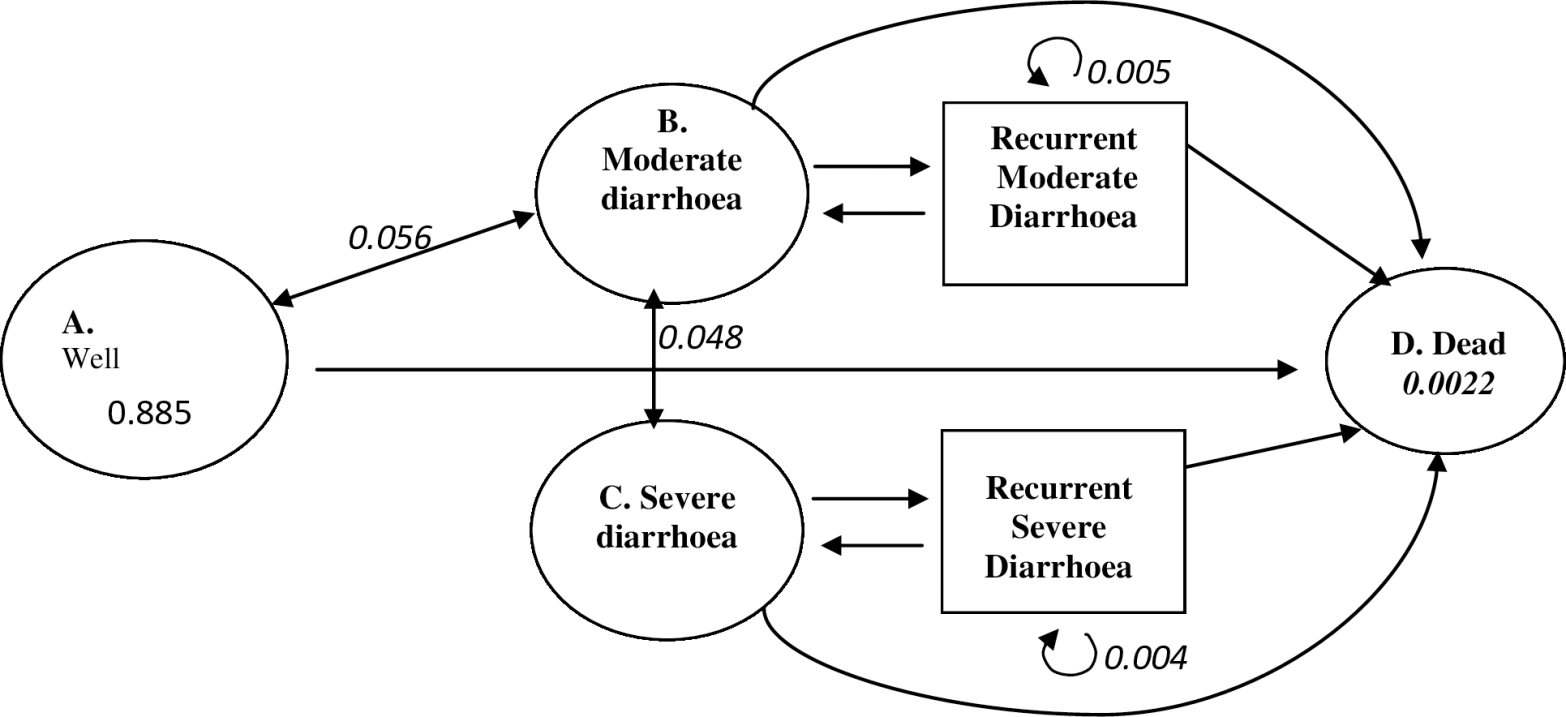
Model figure showing the transition to different health states and their probabilities.

A decision analytic Markov model was used to evaluate the four treatment approaches. The model has four states, namely: well state, moderate state, severe state and death. The starting age in the model was one week. Patients were modelled to start from the well state. They can move to or remain in a health state or die from diarrhoea. See Fig 1. Nigerian specific data were used to construct the model except where not available. The weekly probabilities of staying in any of the health state depended on the risk profile (as shown in Table 2). The transition probability from well to moderate diarrhoea was obtained from age specific incidence for Nigeria [22]. The transition probability of progressing from moderate to severe diarrhoea, recurrent moderate diarrhoea and recurrent severe diarrhoea were obtained from systematic reviews [23], [24]. The transition probability from all cause diarrhoea to death was obtained from a United Nations International Children’s Emergency Fund report on childhood diarrhoea mortality in 2015 in Nigeria [25]. The mortality rate from other causes of disease was calculated from the 2013 Nigerian life table [26]. The model was built using 2013 Microsoft Excel.

**Table 2:** Parameters input and distribution in the Markov model.

Variable	Mean	Distribution	Source
Weekly Transition Probabilities			
Well to moderate diarrhoea	0.056	Beta (0.041–0.093)	[23]
Moderate diarrhoea to severe diarrhoea	0.048	Beta (0.035–0.056)	[24], [25]
Recurrent moderate diarrhoea	0.005	Beta (0.004–0.006)	[24], [25]
Recurrent severe diarrhoea	0.004	Beta (0.003–0.005)	[24], [25]
Remaining well	0.885	Beta (0.511–0.996)	Model
All cause diarrhoea to Death	0.002	Beta (0.001–0.003)	[26]
Relative Risk			
Rotavirus vaccine on all cause of diarrhoea	0.690	Log-normal (0.57–0.85)	[35]
IMCI on Moderate diarrhoea	0.155	Log-normal (0.099–0.255)	[32], [33]
IMCI on Severe diarrhoea	0.153	Log-normal (0.087–0.215)	[32], [34]
Breastfeeding on diarrhoea	0.350	Log-normal (0.58–0.82)	[13]
Cost per treatment course/episode			
Rotavirus vaccine	24.86	Gamma (±25%)	[43]
IMCI Moderate Diarrhoea	11.34	Gamma (±25%)	[41], [42]
IMCI Severe Diarrhoea	26.21	Gamma (±25%)	[41], [42]
Disability weights			
Moderate diarrhoea	0.202	Beta (0.133–0.299)	[29], [30]
Severe diarrhoea	0.281	Beta (0.184–0.399)	[29], [30]
Discount rate			
Cost Utility	3% 3%	N/A (min 0%, max 6%) N/A (min 0%, max 5%)	[21] [21]

N/A: Not applicable

This model approach was preferred because it has the ability to represent repetitive events and it is time dependent. Diarrhoea is a disease whose risk is continuous over time. The administration of the vaccine is time bound (6 to 32weeks) and the disease occurs up to 3 times per year. Representing these in a decision tree for instance will affect the quality of the result.

In the model, we assumed that the children will start from the well state and with each week that passes (Markov cycle), they may remain well; they may experience moderate or severe diarrhoea, they may remain in diarrhoea (non-fatal); or they may die from diarrhoea or other causes not related to diarrhoea. The model assumed that all mothers will breastfeed their children up to 1 year in accordance with the recommendation of WHO and UNICEF and deemed practicable in Nigeria [27], [28]. L-ORS and IVF were usually given until diarrhoea stops or improves but in our model they were given for 3 days while zinc was given for 10 days [6], [10]. The model was designed such that a child will have an average of 3 diarrhoea episodes per year [2]. In the NT approach, we assumed that the mother/caregiver will visit the physician but fail to treat. She will rather administer breast milk to the child.

### Time horizon and discount rate

The Markov cohort model was employed to simulate clinical outcomes and costs during a life cycle of 260 weeks (since most cases of childhood diarrhoea occur between the ages of 0 to 5 years of age) for an estimate of 33 million children under the different alternative intervention scenarios.

The cost and health outcome for the interventions were discounted at a rate of 3% based on the WHO CEA guideline [21].

### Choice of health outcome

Health outcome was presented in disability adjusted life years (DALYs). The DALY calculation was based on the recent Global Burden of Disease 2010 study and used recently updated disability weights [29], [30]. DALYs were calculated for each cycle, accumulated over the model time horizon and then averaged to obtain the DALYs per patient. This was repeated for each diarrhoea management approach.

### Measurement of effectiveness

A comprehensive review indicated that promotion of breastfeeding was one of the most important interventions for controlling diarrhoea among children [13], [31] and thus, it was used as the base case scenario. We calculated the relative risk for moderate diarrhoea from studies in Nigeria that presented treatment success with ORS with a utilization rate of 74.6% [32], [33]. Risk difference between ORS and IVF of 4% was used to calculate relative risk for severe state [32], [34]. A recent systematic review provided the relative risk of rotavirus vaccine on all cause diarrhoea for Nigeria, Ethiopia and Democratic Republic of Congo [35]. We used diphtheria-tetanus-pertussis (DTP3) immunization coverage rate of 70% in Nigeria as a proxy for coverage rate of rotavirus vaccination [36]. The implementation coverage rate of IMCI for diarrhoea (58.6%) was obtained from a Nigerian based study [15]. The relative risk of zinc was obtained from the final estimate of a commentary [37], which analysed three systematic reviews on the effect of zinc supplementation on diarrhoea treatment [38], [39], [40]. The relative risk of breastfeeding on diarrhoea was obtained from the WHO library [13]. The relative risk of zinc and L-ORS on diarrhoea was combined to obtain the resultant relative risk for moderate diarrhoea. The relative risk of IVF and zinc was also combined to obtain the relative risk for severe diarrhoea. Details of input parameters and their distributions are shown in Table 2.

### Estimating resources and cost

Cost was estimated from the payer’s perspective which included direct medical cost (medications cost, health professionals services, hospitalization) [21]. The economic definition of cost based on the concept of opportunity cost was applied in the cost valuation. Cost of medications were obtained from the Nigerian National Health Insurance Scheme (NHIS) drug price list, published in 2005 and 2013 [41], [42]. Cost of ORS, Ringer’s lactate, and retinol were obtained from the 2013 NHIS drug price list while cost of physician consultancy, physician review, nursing service and hospital-stay were obtained from the 2005 NHIS drug price list. Cost of zinc supplement and RV1 were obtained from the 2013 International Drug Price Indicator guide [43].

### Currency, price date and conversion

Cost obtained from the NHIS were adjusted to reflect the future (2016) value using interest rate of 3% (range of 0% - 6%) [21], [44]. For cost obtained from the International Drug Price Indicator guide, the median price was used and adjusted to reflect the 2016 price. Price adjustment entails inflating price using the consumer price index inflation calculator. [45], [46], [47]. Gamma distribution was used to capture the uncertainty inherent in the cost parameter. All costs were converted to 2016 US dollar and a discount rate of 3% was used for all future cost.

### Analytical methods

Treatment course/episode cost for each of the treatment approaches was calculated. Cost for each approach was obtained by summing up the cost components. For each cycle for each treatment approach, cost was obtained by summing up the number of patients with diarrhoea and multiplied by the cost of management and then discounted. The cost from cycle 1 to 260 was then summed up and averaged to obtain the cost to manage a patient (standard cost). The standard cost of each treatment approach was then used to perform the probabilistic sensitivity analysis (PSA).

DALY was also calculated by combining years lived with disability (YLD) and years of life lost (YLL) for each weekly cycle. YLD was calculated as follows: YLD = Number of cases × duration till remission or death × disability weight [29], [48]. The recently updated disability weights were used [30]. Children in the well or asymptomatic were assigned a disability weight of 0 [30]. YLL was calculated as follows: YLL = Number of deaths due to diarrhoea × life expectancy at the age of death [48]^.^ Standard life expectancy (0–4 years) of 57.5 years was obtained from the 2013 Nigerian life table [26]. The DALYs across each cycle was summed and averaged to obtain the standard DALYs which was used in the PSA. DALYs averted was calculated as the difference between the NT DALYs and the DALYs of each of the other interventions.

We identified cost-effective approaches to management of childhood diarrhoea in Nigeria by calculating the incremental costs-effectiveness ratio (ICER) of each of the intervention against the next best effective approach. ICER represents the average incremental cost associated with 1 additional DALY averted. As the threshold for an intervention to be cost-effective is currently still being debated, specifically the traditional 1–3 times GDP per capita used by WHO-CHOICE, the alternative 0.52 times GDP per capita suggested by the University of York was also used in our analysis [49]. In this case, interventions with an ICER below 0.52 times the GDP per capita are considered very cost-effective. The mean ICERs with their 95% confidence interval from the 10,000 iterations were calculated for each intervention.

We performed a univariate sensitivity analysis to know the parameters and assumptions the result was sensitive to using the variables upper and lower limit at 95% confidence interval. For variables without confidence interval like primary cost data +/- 25% was used.

PSA was used to assess simultaneous uncertainty in many variables. This approach is well suited to express overall parameters uncertainty [50]. A total of 10,000 iterations of Monte Carlo simulations was conducted and for each iteration a value was drawn randomly from each distribution and net health benefits calculated [50]. We used cost-effectiveness acceptability (CEA) frontier to illustrate the degree of uncertainty in the estimates. The CEA frontier explored relative efficiency of the interventions, thus showing the likelihood of an intervention being acceptable by the decision-maker. In order words, the CEA frontier illustrated the probability of any intervention being optimal compared to all other competing alternatives. Sixty-five (65) iterations of simulations were conducted for different willingness-to-pay threshold ratio. For each iteration, the probability that the cost-effectiveness of any intervention being optimal compared to other competing interventions was calculated for all the alternative interventions from the NMB [50].

## Results

### Incremental cost and outcome

Cost analysis over a life cycle of 260 weeks showed that IMCI approach has the least cost per patient ($9.08) after NT ($5.20). Rotavirus vaccine + IMCI approach has the highest cost ($32.32) followed by the rotavirus vaccine approach ($20.61). In all, NT has the least cost when compared to the other interventions. The NT approach has no DALY averted (since it was used as baseline). The rotavirus vaccine + IMCI approach averted the highest DALYs. Table 3 shows details of the result.

**Table 3:** Results showing cost, effect and ICER per patient.

Interventions	Cost ($)	DALYs Lost	Incremental Cost	Incremental DALYs averted	ICER with 95% confidence ($/DALYs averted)	Remark
No treatment	5.20	1.62	0.00	0.00	0.0	—
Rotavirus vaccine	20.61	1.59	—	—	—	Stg Dom*
IMCI	9.08	0.77	3.88	0.85	4.6 [ᶲ – 11.5]	
Rotavirus vaccine + IMCI	32.32	0.48	23.24	0.29	80.1 [70.6–97.1]	

* Stg Dom: Strongly Dominated

ᶲ: - 1.3

IMCI: Integrated Management of Childhood Illness

As shown in Table 3, the rotavirus vaccine approach was strongly dominated and was excluded in ICER analysis. the ICER of the IMCI and rotavirus vaccine + IMCI interventions were less than 0.52 times the GDP per capita [49] of Nigeria which was US$2,177.99 in 2016 [51]. Thus, IMCI and rotavirus vaccine + IMCI approaches were all very cost-effective. IMCI approach had the smallest ICER and is cost-saving relative to the rotavirus vaccine approach. The result also showed that rotavirus vaccine + IMCI approach had the highest ICER below the threshold and thus the most cost-effective.

### Characterizing uncertainty

**Fig 2:**
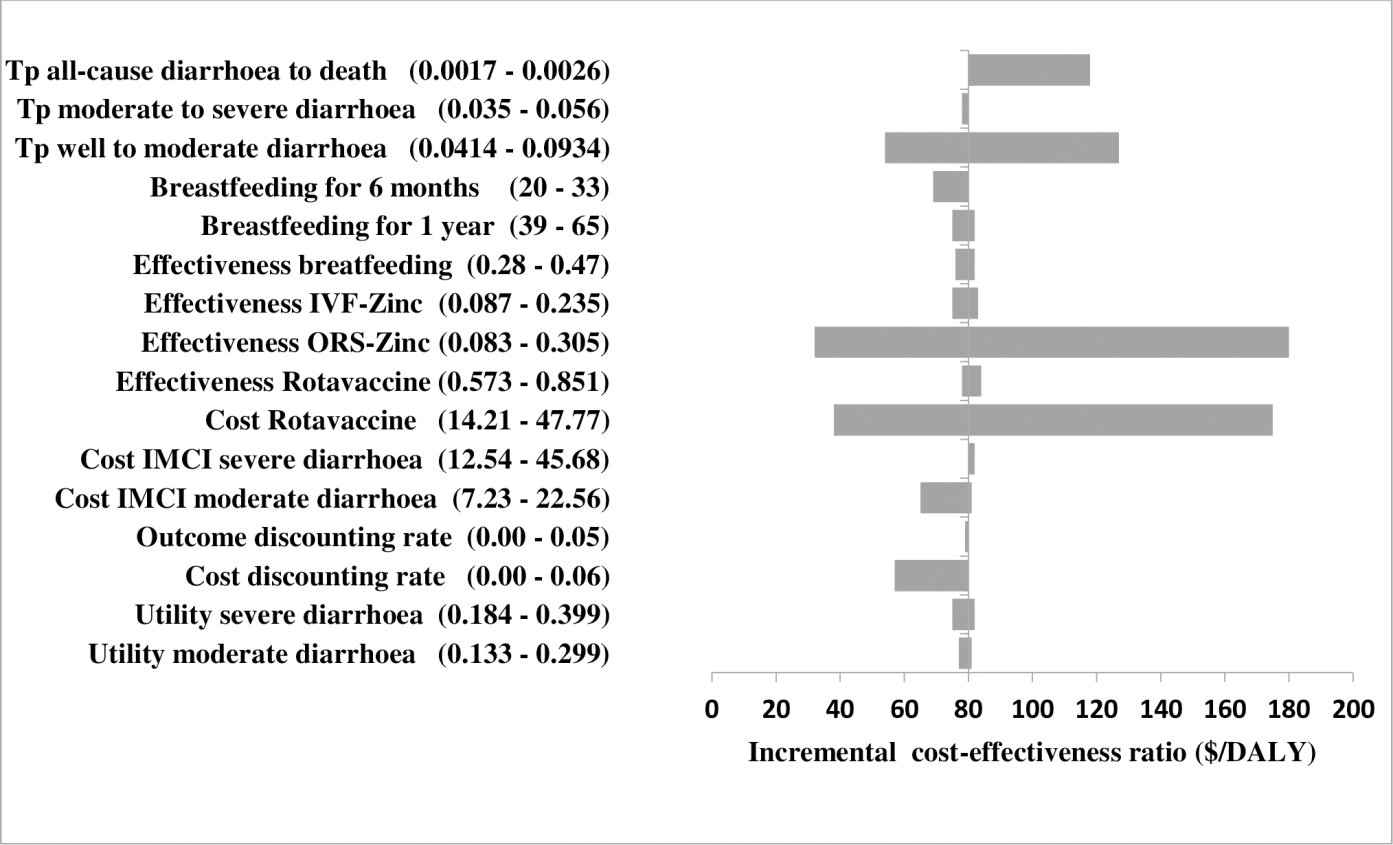
Tornado diagram showing the uncertainty impact of key parameters on the ICER result of the most cost-effective approach. Tp: Transition Probability Left: Higher limit ICER values Right: Lower limit ICER values.

The univariate sensitivity analysis showed that the effectiveness of ORS-zinc was the most influential parameter followed by cost of rotavirus vaccine. At lower limit of ORS-zinc effectiveness (0.083) the ICER increased from $80/DALY to $180/DALYs while at higher limit (0.305) the ICER reduced to $32/DALYs. Other parameters with significant influence on the result of the model were transition probability from well to moderate diarrhoea, and transition probability from all-cause diarrhoea to death. Outcome discount rate and cost of IMCI severe diarrhoea had insignificant effect on the result. Details are shown in Fig 2.

**Fig 3:**
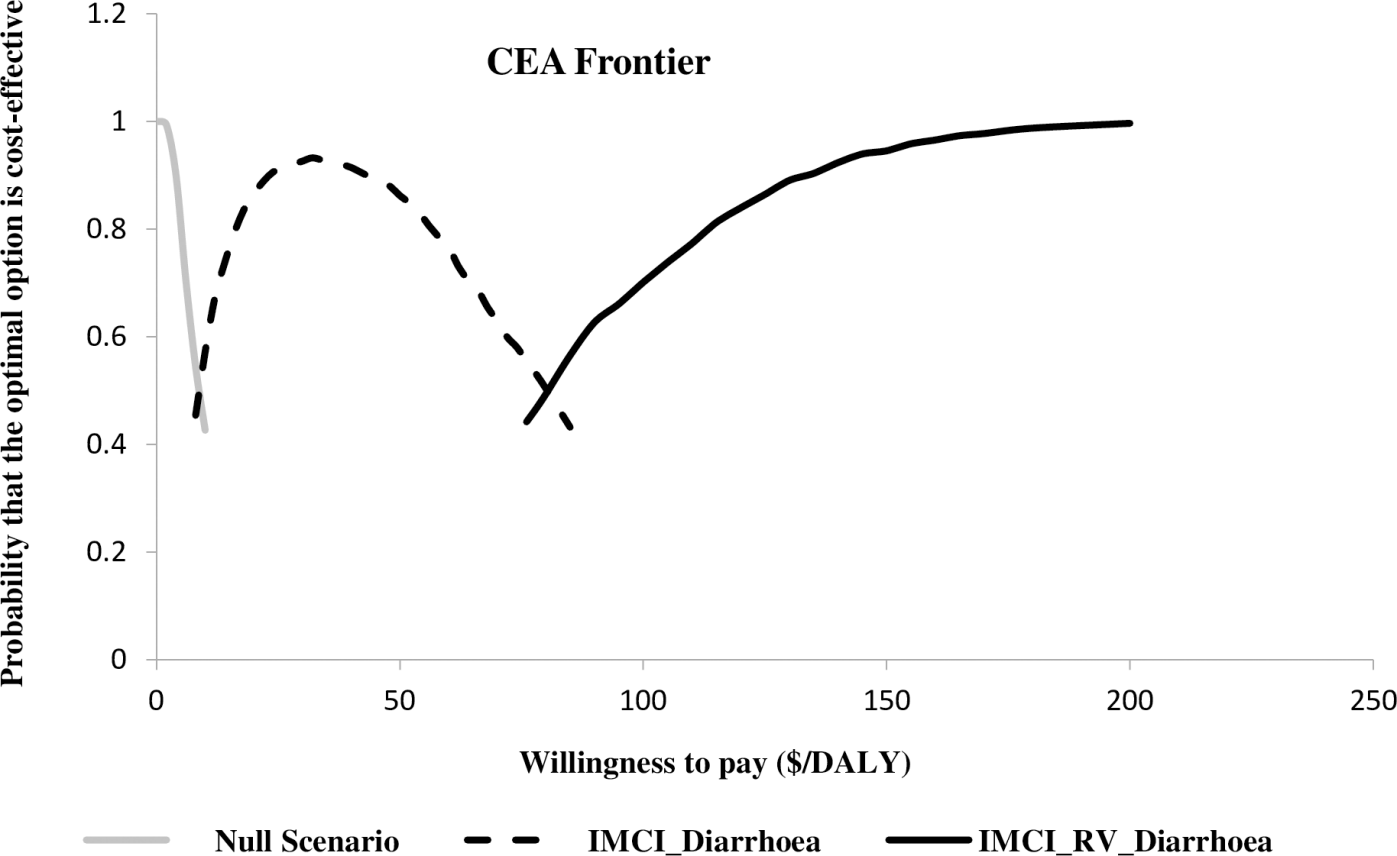
Cost-effectiveness acceptability frontier showing the decision uncertainty surrounding the optimal choice.

Fig 3 further illustrates the relative efficiency of the interventions in optimizing health care resources allocation. Under parameters uncertainty and over some willingness-to-pay values, the CEA frontier illustrated which intervention for management of childhood diarrhoea had the highest probability of being cost-effective. In other words, it shows the decision uncertainty surrounding the optimal choice. The NT approach had the highest probability of being cost-effective at no willingness-to-pay value. When the payer is willing to provide at least $8 to avert a DALY, the IMCI approach had the highest probability of being cost-effective. Rotavirus vaccine + IMCI had the highest probability of being cost-effective when the payer is willing to pay above $80 to avert one extra DALY over IMCI. Rotavirus vaccine alone was dominated as it showed a zero probability of being cost-effective at any willingness to pay.

## Discussion

This study aimed to identify cost-effective approaches to childhood diarrhoea management in Nigeria using a decision analytical model. The study compared the ICER of three approaches to the 0.52 times GDP/capita threshold by Woods et al. [49] in order to determine which approaches were cost-effective. The acceptability frontier was also used to check the relative efficiency between the interventions. IMCI approach was cost-effective while rotavirus vaccine + IMCI approach was a more cost-effective approach. IMCI and rotavirus vaccine + IMCI approaches were more efficient than rotavirus vaccine alone in optimizing health care resources allocation and thus rotavirus vaccine alone was deemed dominated. Based on the univariate analysis result, uncertainty surrounding effectiveness of ORS-zinc; cost of rotavirus vaccine and transition probability from well to moderate diarrhoea had the highest effect on the result of the cost-effectiveness analysis. Therefore obtaining more precise information about these most influential parameters would be worthwhile in order to inform the decisions.

The IMCI for diarrhoea provides simple and effective methods to manage diarrhoea which is a leading cause of illness and mortality in young children. The implementation guideline of IMCI promotes evidence-based assessment and treatment using syndromic approach that support the rational, effective and affordable use of drugs [14]. The guideline involves checking the child’s nutritional status, certain symptoms like fever, sunken eyes, lethargic or unconscious; teaching parents how to give treatment at home; assessing a child’s feeding and counseling to solve feeding problem; and advising parent when to return to clinic. Basically, this approach is designed for use in outpatient clinical settings with limited diagnostic tools, limited medications and limited knowledge and skills to practice uncomplicated clinical procedures [14]. Since IMCI is a cost-effective approach, payers like NHIS and other health maintenance organisations in Nigeria should ensure the spread of IMCI in health facilities and ensure wide uptake of IMCI by all nursing mothers. As an integrated approach, IMCI will impact positively on other childhood diseases and the general wellbeing of children and thus will be a worthwhile approach to promote. Unfortunately, the awareness and uptake of IMCI in Nigeria is still not optimal [52]. In a study in Ibadan, Nigeria, only 50.9% of nurses had a high positive attitude towards the IMCI strategy [52]. There is need to step-up training coverage on IMCI for Nigerian health workers who will educate mothers and caregivers. Radio jingles and television adverts will help facilitate the awareness, knowledge and practice at home.

For additional benefit, the inclusion of rotavirus vaccine to the Nigerian national immunization program should be considered since its combination with diarrhoea treatment using IMCI was more cost-effective from our findings. Nigeria is eligible for Global Alliance for Vaccinations and Immunisations (GAVI) support and in principle should be able to exploit the low vaccine cost offered by GAVI to procure the vaccine. Unfortunately, GAVI support for Nigeria was suspended due to systemic weaknesses regarding the operation of controls and procedures used to manage GAVI cash-based support [53]. It is of uttermost importance that the National Primary Health Care Development Agency work with urgency to remedy the weaknesses in the operation of controls and procedures used to manage GAVI cash-based support.

Our analyses have limitations which are governed by data availability and our assumptions. Certain data used in the model were not specific to Nigeria. Examples include the relative risk ratio of RV1 and some transition probabilities. Though these data were obtained from sub-Saharan Africa’s studies and systematic reviews, they may not represent a true picture for Nigerian scenario. More importantly, for a disease like diarrhoea, the inclusion of outbreaks of other causes of diarrhoea like bacteria (Clostridium. difficile, E.coli, shigella etc), parasites (giardiasis), food allergy etc in addition to rotavirus would have been relevant. This static model will underestimate the indirect benefit of rotavirus vaccine and therefore underestimate its cost-effectiveness [54]. Furthermore, the perspective of our analyses was the payer’s perspective and not the societal. Thus, indirect cost like cost of transportation, extra-nutritional child requirements, cost of diapers which would have been incurred by the mother/caregiver were excluded in our analyses. Taking such cost into consideration would have yielded a more practical cost effectiveness result.

Similar to our result, some studies established rotavirus vaccine paired with diarrhea treatment as the most cost-effective option. For instance, a recent study in Ethiopia found that diarrhoea treatment paired with rotavirus vaccine is more cost-effective than diarrhoea treatment alone [55]. Another Tanzanian based study showed that rotavirus vaccine provided as a package with diarrhoea treatment is highly cost-effective compared to the implementation of diarrhoea treatment alone or only providing Rotavirus vaccine [19]. However, the cost of rotavirus vaccine assumed within this analysis was higher compared to other studies. The reason for the difference in cost of vaccine between our study and others is because rotavirus vaccine is currently not subsidized in Nigeria.

In conclusion, our model suggests that in the Nigerian context, inclusion of rotavirus vaccination to IMCI for diarrhoea management was the most cost-effective approach to childhood diarrhoea management. IMCI for diarrhoea should be highly advocated in Nigeria since it is cost-effective. Training programs for mothers, antenatal mothers and radio jingles may be necessary to increase practice of IMCI at homes. Nigerian government should consider rotavirus vaccination as part of national programme of immunization as it could provide additional benefit to diarrhoea management. Although our findings suggest that addition of rotavirus vaccine to IMCI for diarrhoea was the most cost-effective approach, there may be need for further vaccine demonstration studies or real life studies to establish the actual cost-effectiveness of the vaccine in Nigeria.

## Supporting information

S1 Appendix Model design showing input parameters, analyses and results. (XLSM)
